# The content comparison of health-related quality of life measures in heart failure based on the international classification of functioning, disability, and health: a systematic review

**DOI:** 10.15171/jcvtr.2019.29

**Published:** 2019-08-13

**Authors:** Mahdi Moshki, Abdoljavad Khajavi, Farveh Vakilian, Shima Minaee, Haydeh Hashemizadeh

**Affiliations:** ^1^Department of Health Education and Promotion, School of Health; Social Development and Health Promotion Research Center, Gonabad University of Medical Sciences, Gonabad, Iran; ^2^Department of Community Medicine, School of Medicine, Gonabad University of Medical Sciences, Gonabad, Iran; ^3^Department of Cardiology, Preventive Atherosclerotic Research Center, Imam Reza Hospital, Faculty of Medicine, Mashhad University of Medical Sciences, Mashhad, Iran; ^4^Department of Cardiovascular Diseases, Razavi Hospital, Mashhad, Iran; ^5^Social Development and Health Promotion Research Center, Gonabad University of Medical Sciences, Gonabad, Iran

**Keywords:** Quality of Life, Health Status, Heart Failure, Content Analysis, Systematic Review

## Abstract

***Introduction:*** Due to the necessity of assessing the health-related quality of life (HRQOL) in heart failure (HF) and the increased use of the International Classification of Functioning, Disability, and Health (ICF) for making a content comparison of measurement instruments, the present study aimed to evaluate the relationship between the instruments and ICF. To this aim, the disease-specific HRQOL instruments in HF were identified, and then psychometric properties and content comparison of included instruments were conducted by linking to ICF.

***Methods:*** Disease-specific HRQOL instruments in HF were identified through a comprehensive and systematic search strategy. Then, the psychometric properties of included instruments were determined, and their contents were analyzed and compared based on the ICF coding system. In addition, each instrument was independently linked to ICF by two researchers based on standardized linking rules, and finally their degree of agreement was assessed by the Cohen’s kappa coefficient.

***Results:*** Ten instruments including a total of 247 items and 417 concepts were linked to 124 different ICF categories. Further, 39 (31.5%), 65 (52.5%), 13 (10.4%), and 7 (5.6%) categories were linked to body function, activity and participation, environmental factors, and body structure, respectively. According to the content analysis approach and psychometric properties, the appropriate measurement instruments were Kansas City Cardiomyopathy and Minnesota living with HF questionnaires, respectively.

***Conclusion:*** Content comparison provides researchers with valuable information on the instrument heterogeneity and overlapping, which results in selecting the most appropriate measurement instrument based on a specific clinical context.

## Introduction

The effective management of chronic diseases such as heart failure (HF) has increased the concern of health systems due to the aging population of the world. These services reduce the symptoms of the disease and improve the quality of life (QOL). HF is considered as a rapidly growing public health issue which has influenced 5.8 million people in the United States, and 650 000 new cases were annually reported.^1^ In Iran, the patients with HF allocate 3.3% of the population.^2^ The patients have experienced physical and emotional symptoms such as dyspnea, fatigue, edema, sleep disturbances, depression and chest pain, leading to the disturbance in their QOL.^3^ HF has a more complex nature compared with other chronic diseases such as cancer, diabetes, and chronic obstructive pulmonary disease and has a more unpredictable outcome.^4^ Thus, patients need frequent, long-term, and costly hospitalization.^5^

The significance of patients’ perspective resulted in developing patient*-*reported outcomes measurement information system. Todays, the implementation of health-related quality of life (HRQOL) instruments in clinical trials and clinical practices is increasing.^6^ HRQOL instruments focus on the activity and participation of patients as their main component. These instruments not only can measure and compare performance and health status during the course of the disease, but also evaluate the performance and health in various populations and different clinical practices.^7^ Due to the significance of the symptoms and functional limitations caused by HF, using HRQOL measurement instruments seems necessary.

Choosing the appropriate outcome measure is an important and difficult task for researchers and physicians. Some recent systematic reviews about HF-specific HRQOL instruments were compared with the psychometric properties of measurement instruments.^7,8^ However, the detailed content comparison of HRQOL instruments in HF has less been addressed in the literature. Ideally, the measurement should be conducted based on recognized theoretical foundations, while original articles describing the development of a measure have been less highlighted. Content analysis is considered as one method for filling the existing.^9^ The World Health Organization (WHO) introduced ICF as a global framework for comparing HRQOL measurement instruments based on content. In addition, ICF was developed as a multipurpose classification framework in order to provide a common and universal language for describing a wide range of HRQOL instruments.^10^ However, the conducted search for content comparison of HRQOL instruments in HF was not successful. Therefore, the present study sought to identify the disease-specific HRQOL instruments in HF and compare the psychometric properties and contents of the included instruments by linking to ICF.

## Methods

### Design of the study

The present study is a specific systematic review type in the field of measurement instruments in order to compare disease-specific HRQOL instruments based on contents by linking to ICF. This systematic review was performed after developing protocols and its registration.^9^ The systematic review was registered in International Prospective Register of Systematic Reviews (PROSPERO) (No. CRD42015025380; http://www.crd.york.ac.uk/PROSPERO/display_record.asp?ID=CRD42015025380). First, disease-specific HRQL instruments in HF were identified through a comprehensive and systematic search strategy on MEDLINE, CINAHL and Scopus databases from January 1960 to January 2017. Then, ten instruments were included in the systematic review. Finally, the psychometric properties and content comparison of the instruments were performed.

### Step one

#### Search strategy

In order to find the existing HF-speciﬁc HRQOL instruments, a comprehensive and systematic search was done using free text and MeSH Terms in the International databases MEDLINE (via PubMed), CINAHL (via EBSCO) and Scopus (via Elsevier). The search terms in the field of HF included “heart failure”, “chronic heart failure”, “severe heart failure”, “congestive heart failure”, “cardiomyopathy”, and “left ventricular disease”, while the QOL consisted of “quality of life”, “health-related quality of life”, and “health status”. In addition, the filter provided by PubMed for searching the records of Patient-Reported Outcome Measures (PROMs) was applied (see Supplementary file 1).^11^ Since citation searches are more sensitive than keyword searches in the conducted studies on measuring instruments, citation was searched in this study.^9^ Further, the articles were searched by hand in the Patient-Reported Outcome and QOL Instruments Database (PROQOLID) (www.proqolid.org). Finally, the systematic reviews published in the field of HF specific QOL instruments were use.^7,8^

#### Inclusion and exclusion criteria

The population included adults with HF. Therefore, non-adult subjects as well as the patients with other cardiac disorders such as coronary artery disease, myocardial infarction, pectoral angina, atrial fibrillation, peripheral artery disease, stroke, and vascular diseases were excluded from the study. The studies related to development, validation, reliability, and responsiveness were included in the present research. However, linguistic validation studies as well as all the sign-related instruments, disease severity measures, disease control measures, and cardiac physiology were excluded from the study. The outcome measure of this study included QOL, HRQOL, and health status in HF. It is worth noting that accessing full English texts during January 1960-2017 for each instrument was essential in the present study. Finally, 10 instruments were included after excluding the articles which were not related to the study objectives. *Screening*

#### Screening

**Figure 1:**
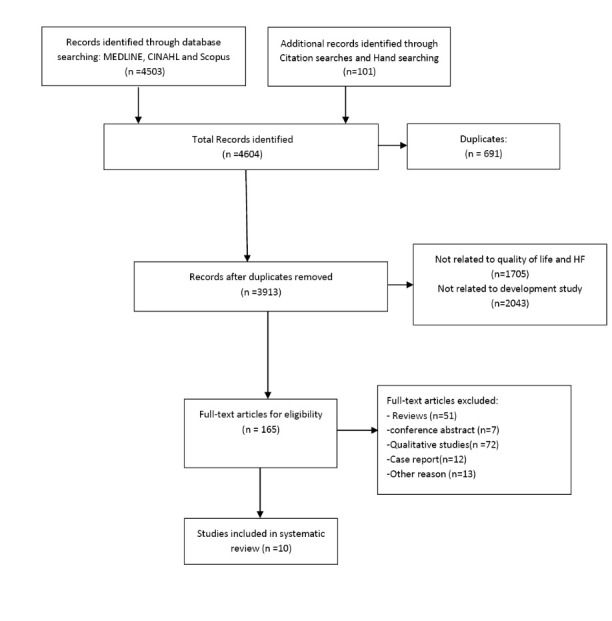
PRISMA flow diagram.

The retrieved literature was screened in four steps. First, the whole retrieved literature was added to EndNote (Thomson Reuters, Philadelphia, PA, USA) in order to facilitate the identification and remove the duplicates. Second, two reviewers (M.M, A.K) evaluated the eligibility of all the retrieved literature separately based on their titles and abstracts by using the inclusion checklist which was set according to the inclusion and exclusion criteria. Any disagreement between the two reviewers was considered to choose the articles and, if necessary, a third reviewer (S.M) was asked for her comments. Third, the full texts of collected articles were reviewed for eligibility by the two reviewers (M.M, A.K). Again, the third reviewer was asked for clarification in the case of any disagreement between the two reviewers. Finally, PRISMA search flowchart was used to include and exclude the studies (Figure 1).

#### Step two

#### ICF

**Figure 2:**
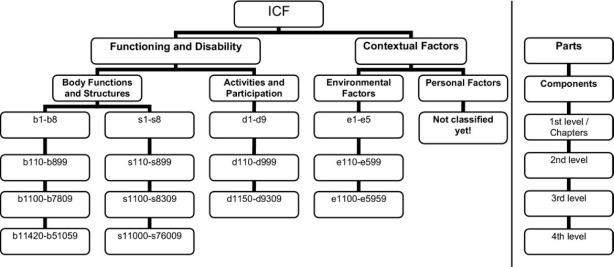
Structure of the International Classification of Functioning, Disability and Health (ICF) within the chapters (i.e., the ﬁrst item level), categories can be discriminated (i.e., 2nd to 4th item level). Reprinted with permission from Velstra et al.^12^

As a standard framework, ICF includes health-related dimensions, and a comprehensive description of human functioning. ICF has two parts, each including two separate components. The first part covers functioning and disability, and includes the components of body functions (b), body structures (s), and activities and participation (d). The second part covers contextual factors and involves the components of environmental factor (e) and personal factor (pf).^12^ Body functions refer to the physiological and psychological‏ functions of the body system. Body structures represent the anatomical parts of the body such as organs, limbs, and their components. Activities and participation include a full range of life areas such as learning, interpersonal interactions, and employment. Environmental factors consist of the physical, social, and attitudinal environment in which people live. Personal factors, which are not yet classified in ICF, form the particular background of individual’s life by considering non-health-related parts such as gender, race, age, fitness, lifestyle and habits.^13^ In the ICF classification, the letters introducing each component (b, d, s, and e) are followed by a numeric code (one-digit) which represents the chapter number or the first level. Then, the second level (two-digit) is followed by the third and fourth levels (each with a one-digit number). Therefore, ICF levels are arranged in a stem-branch-leaf (Figure 2).^14,15^

#### Standardized linking rules

Before starting theprocess of linking the outcome measures to ICF categories, it was essential to identify all meaningful concepts in each item of the health status measure. The information related to each construct should have been linked to ICF when a single item contained different constructs. If the response options of each item had meaningful concepts, they would be linked to ICF. The time interval for each item such as “Last week,” should not have been linked. Further, both the concept and examples should have been linked when a meaningful concept of an item was explained through examples. Before linking meaningful concepts to ICF categories, it was necessary to have good knowledge of ICF taxonomical and conceptual fundaments, chapters, domains, and categories. Each meaningful concept should have been linked to the most precise ICF category. Furthermore, the additional information was named in ICF when the content of a meaningful concept was not clearly identified in ICF categories. If the information provided by the meaningful concept were not enough to make a decision on its linking to the most precise ICF category, it would be linked to not-definable category (nd). Additionally, the meaningful concept should have been considered as a personal factor when a meaningful concept was related to personal factors and was not available in ICF. If a meaningful concept were not available in ICF without relating to personal factors clearly, this meaningful concept would be considered as not covered (nc). Finally, a meaningful concept should have been considered as a health condition (hc) when it referred to diagnosis or health condition.^13^

#### Data analysis

The data were analyzed based on systematic content analysis and linking to ICF. In order to provide the quality of evaluation, each of the ten instruments were separately linked to ICF using the data extraction instruments based on 10 linking rules developed for this purpose and done by two of the researchers (F.V, H.H). Their degree of agreement or the reliability of the linking process was confirmed according to Cohen’s kappa coefficient with IBM-SPSS-23. A third person would be consulted (S.M) when the two researchers failed to have a consensus on ICF linking concepts. The number of items was reported in each instrument and their containing concepts were linked to the ICF components (b, d, e, s, nc, pf). The frequency of ICF categories represented the concepts of each instrument and, therefore, became the basis for descriptive analysis and content comparison.

## Results

Through the search strategy, ten HF-specific HRQOL measurement instruments including Chronic Heart Failure Assessment Tool (CHAT),^16^ Cardiac Health Profile congestive heart failure (CHP-CHF),^17^ Chronic Heart Failure Questionnaire (CHFQ)^18^, Kansas City Cardiomyopathy Questionnaire (KCCQ),^19^ Left Ventricular Disease Questionnaire (LVDQ),^20^ Minnesota Living with Heart Failure Questionnaire (MLHFQ),^21^ Quality of Life in Severe Heart Failure Questionnaire (QLQ-SHF ),^22^ Mac new,^23^ San Diego,^24^ and Heart QOL^25^ were identified. Table 1 indicates the **c**haracteristics and psychometric properties of the disease-specific HRQL instrument for HF patients. The reliability of all instruments were confirmed except SDHFQ. The responsiveness was only reported for MLHF, KCCQ, CHFQ, LVDQ, MacNew, and Heart QOL. As shown in Table 2, the results of linking process were confirmed by calculating Cohen’s kappa coefficient with a confidence level of 95%. The estimated Kappa Cohen coefficients ranged between 0.82 and 0.93, indicating an acceptable agreement. Among the ten instruments, 417 concepts were identified. CHAT (67 concepts) was considered as the highest and Heart QOL (22 concepts) was the lowest number of concepts. All HRQOL instruments were covered by body functions. Activities and participation were not covered by CHFQ. Additionally, the environmental factors were not covered by CHFQ, QLQ-SHF, MacNew, and Heart QOL. In addition, the body structure was not covered by CHFQ, CHAT, QLQ-SHF, MacNew and Heart QOL. Most concepts or 222 ‏53.3)%) were linked to the Body functions, and 134 concepts (32.2%) to the activity and participation. However, 28 concepts (6.7%) were linked to the environmental factors and 21 (5%) to the body structures (Table 3). Further, the linkages of concepts were represented through some tables such as the linkage to the body functions (Table S1), activity and participation (Table S2), environmental factors (Table S3), and body structures (Table S4). However, ICF was not differentiated in some cases. Therefore, many items or concepts of HRQOL instruments were merely linked to one category like emotional function (b152), which was linked to a large number of feelings such as *concern, tension, worried, frightened, annoyed/angry, wonder, dependent, anxious, afraid, depressed, irritated, sad, uneasy, down in the dumps, upset, relaxed, under tension, discouraged, happy, satisfied, pleased, restless, tense, uptight, frustrated, real nuisance, feeling ill, and limited*.

**Table 1:** Characteristics and psychometric properties of the disease-specific HRQOL instruments for HF

**Instruments**	**Author/ year/ Country**	**Setting of development**	**Construct validity**	**Reliability**	**Responsiveness**
MLHF/MLHFQ/LHFQ/LiHFe ‏)Minnesota Living with Heart Failure Questionnaire‏(	Rector et al, 1987, USA	83 patients (84% male and 16% female) with Congestive Heart Failure	Correlated with NYHA	Test re-test, Internal consistency: Physical (α=0.92) Emotional/psychological (α=0.87).	Sensitive to change
KCCQ ‏)Kansas City Cardiomyopathy Questionnaire‏(	Green et at, 2000, USA	129 patients (69% male and 31% female, 70 stable, 59 decompensated) with congestive heart failure	Correlated with NYHA, 6MWT, MLHFQ,SF-36	Test re-test,Internal consistency: 1. Physical limitation (α =0.90), 2. Symptoms (α=0.88), 3. Self-efficacy (α=0.62), 4. QoL (α=0.78), 5. Social limitation (α=0.86)	It was more responsive to major clinical change than the Rand SF-36 and the MLHFQ
CHFQ ‏)Chronic Heart Failure Questionnaire‏(	Guyatt et al., 1989, Canada	88 patients (70.5% male and 29.5% female) with Chronic Heart Failure	Convergent and discriminant validity and the factor structure has been supported.	Test-retest Internal consistency: α =0.83–0.95.	Sensitive to different severities of CHF
LVDQ LVD-36 (Left Ventricular Dysfunction Questionnaire‏(	O'Leary & Jones, 1998, UK	60 patients (76.6% male and 23.4 female) with Chronic left ventricular dysfunction	Correlated with Rand SF-36 and MLHFQ	An intraclass correlation coefficient between baseline and repeat questionnaire scores was calculated, The Kuder-Richardson coefficient in both cases was 0.95.	Measures changes in health status when the questionnaire was repeated after 6 months.
QLQ-SHF ‏)Quality of Life in Severe Heart Failure Questionnaire‏(	Wiklund et al, 1987, Sweden	51 patients(64.7% male and 35,3% female) with severe heart failure	Correlated with SIP,Construct validity is acceptable for the domains of Emotional/cognitive and life satisfaction.	Test re-test α =0.88	-
CHP-CHF ‏)Cardiac Health Proﬁle congestive heart failure‏(	Mannheimer et al, 2007, Sweden	83 patients (80% male and 20% female) with chronic heart failure	Correlated with MLHFQ	-	-
CHAT ‏)Chronic Heart Failure Assessment Tool‏(	Dunderdale et al, 2008, UK	233‏ patients(69.5% male and 30.5% female) with chronic heart failure	Correlated with each of the SF-36 domains, except vitality and mental health, and between the CHAT and all aspects of the MLHFQ	α for each factor was greater than 0.8	-
MacNew (ex-QLMI – Quality of Life after Myocardial Infarction)	(Lim et al, 1993; Valenti et al, 1996) Australia	63 patients(90% male and 10% female) MI	Correlates with SF-36 and MLHFQ.	Internal consistency and intraclass correlation coefficients ≥0.73	Good results for responsiveness post-intervention.
SDHFQ (San Diego Heart Failure Questionnaire)	Shabetai, 1983, USA	No details	Correlated with MLHQ	No details	Failed to differentiate
Heart QOL	Oldridge et al, 2002 to 2011, five regions	6384 patients: angina, n=2111, 33.1%; MI, n=2351, 36.8%; ischemic heart failure, n=1922, 30.1%	Correlated with SF-36; discriminative validity was confirmed with predictor variables: health transition, anxiety, depression, and functional status	α ≥0.80	Sensitive to change following either percutaneous coronary intervention and cardiac rehabilitation

**Table 2:** Percentage agreement between reviewers

	Estimated kappa coefficient	95% Bootstrapped confidence intervals
Component	0.82	[0.71- 0.87]
Chapter 1^st^ level	0.88	[0.82- 0.94]
2^nd^ level	0.93	[0.87- 0.96]
3^rd^ level	0.91	[0.88- 0.95]
4^th^ level	0.9	[0.86- 0.96]

**Table 3:** Heart failure-specific HRQOL instruments: number of identified concepts for all instruments

** **	**MLHFQ**	**KCCQ**	**CHFQ**	**LVDQ**	**QLQ-SHF**	**CHP-CHF**	**CHAT**	**MacNew**	**San Diego**	**Heart QOL**	**Total** **No. (%)**
Number of item	21	23	16	36	26	10	46	27	28	14	247
Number of concepts	38	53	23	51	27	40	67	30	66	22	417
Concept link to ICF component											
Body Functions (b)	13	20	21	31	17	27	29	22	32	10	222 (53.3)
Activity and Participation (d)	15	21		15	9	8	32	6	16	12	134 (32.2)
Environmental Factors (e)	6	4		2		2	5		9		28 (6.7)
Body structures (S)	2	7		1		2			9		21 (5)
Concept not covered by the ICF (nc)	2		1	1	1		1	2			8 (1.9)
Personal factor (pf)		1	1	1		1					4 (0.9)

Further, 124 different ICF categories were used to address the contents of the ten existing instruments including 39 (31.5%) for body functions, 65 (52.5) activity and participation, 13 (10.4%) environmental factors, and 7 (5.6%) categories for body structure. The broadest bandwidth was associated with the instrument CHAT. Energy level (b1300) were covered by all the instruments except Mac new. The emotional function (b152) was covered by all the instruments except San Diego. The respiration rate (b4400) had been addressed by all of the instruments except CHFQ. In the category related to the activities and participation, regarding learning and applying knowledge (d1), only MLHFQ, CHP-CHF, and CHAT addressed on focusing attention (d160). QLQ-SHF was the only instrument containing the item of making decisions (d177). General tasks and demands (d2) was covered by all the instruments other than MLHFQ, KCCQ, CHFQ, San Diego and Heart QOL. Conducting daily routines (d230) was only addressed by CAHT and QLQ-SHF, with CHAT possessing the highest frequency. Mobility (d4) was not covered by CHFQ and CHP-CHF, while other instruments had addressed this factor. In mobility, climbing was more emphasized than walking. The self-care dimension (d5) was covered only by MLHFQ, KCCQ, LVD-36 and CHAT, which was covered by CHAT and KCCQ most frequently. The domestic life (d6) was addressed by MLHFQ, KCCQ, LVD-36, QLQ-SHF, CHAT, San Diego, and Heart QOL. Furthermore, MLHFQ, KCCQ, CHP-CHF, CHAT, Mac new, and San Diego contained interpersonal interactions and relationships (d7). In major life areas (d8), only MLHFQ and CHAT covered the employment dimensions (d850, d8502). Community, social, and civic life (d9) was addressed by all instruments other than QLQ-SHF, CHFQ, MacNew, and San Diego.

Six instruments including CHAT, CHP-CHF, KCCQ, LVD-36, MLHFQ, and San Diego addressed environmental factors, while MLHFQ, KCCQ, CHAT, and San Diego covered environmental factors with more details than CHP-CHF and LVDQ did. In addition, MLHFQ, KCCQ, and CHAT covered the category support and relationships (e3) including support and relationships with immediate family (e310), extended family (e315), friends (e320) and health professionals (e355). Further, five instruments such as KCCQ, MLHFQ, LVD-36, CHP-CHF, and San Diego covered the body structure, among which San Diego and KCCQ had the greatest coverage. Regarding the body structure, more attention was paid to the structure of the lower leg (s7501) and the structure of ankle and foot (s7502). Personal factors were only available in KCCQ (lifestyle), CHFQ (personal Life), LVD-36 (my Lifestyle), and CPH-CHF (your outlook on life). A total of 8 concepts could not be linked to ICF and were marked as non-coverable (nc) such as a burden on the family and friends and the side eﬀects of treatment (MLHFQ), a burden on others (CHFQ), becoming frail or losing one’s credit with others (LVD-36), meaningless existence (QLQ-SHF), the side effects of medication (CHAT), a burden on others, and the excluded ones (MacNew).

## Discussion

Since the content of the items in the instruments can be linked to ICF with the exception of a few cases, the ICF could be used as a standard framework for comparing the content of HF-specific HRQOL measurement instruments systematically. In addition to identifying the instruments, the current study could provide new information on the content of these instruments based on ICF. The instruments identified in this systematic review were different in terms of the number of concepts and categories. Thus, 124 different categories of ICF were used to assess the content of these 10 instruments. The results of the content comparison of the outcome measures in HF indicated that these instruments focused on the assessment of body function, and activity and participation, and failed to address the environmental factor, body structure, and personal factor as much as they should. Therefore, in designing a new instrument, it seems necessary to consider the items related to environmental factors (physical, emotional and economic), personal (personality and motivation), and physical structure. The results from the content comparison of multiple item measurement instruments indicated that these instruments overlap in the components related to body function, and activity and participation. However, none of these items could fully cover ICF components.^14^

In the present study, some‏ of the ICF categories were not specific enough. For example, the emotional function (b152) was not specific enough with respect to the items of the instruments, which seems to be inefficient in this regard. Environmental factors were rarely presented in this study and other similar studies.^26^ Therefore, adding items such as the family’s attitude, significant others, support at work, life satisfaction, work performance, and disability to the new instruments can be helpful. Overlay MLHFQ, KCCQ and LVDQ had more coverage of ICF component, and their construct validity, reliability, and responsiveness were confirmed. Additionally, KCCQ was more responsive to important clinical change in HF patients than the Rand SF-36 and the MLHFQ.^27^ San Diego is not considered as an appropriate instrument due to the lake of good psychometric properties.

While selecting HRQOL instruments for a specific purpose, the first question raised is “What should be measured in what population, and with what type of intervention?”^28^ The second question is “Which instrument should we use?”^6^ In different situations, various tools may be used based on the research question. For example, if the researchers are willing to evaluate only body functions component, the CHFQ is more appropriate because of both good coverage and psychometric properties. On the other hand, as CHFQ fails to cover other ICF components, it is not regarded as a suitable tool for this purpose. McNew has been widely used in HF patients, although it is more appropriate in myocardial infarction. Thus, it is not justifiable to use when other HF instruments are available. The LVDQ covers ICF components although it may not be appropriate since this instrument has a dichotomous response option and researchers may be more willing to use Likert scale. Since the reliability of CHFQ, KCCQ, and MLHFQ is more than LVDQ, its use may not be logical. The Heart QOL is considered as an instrument which was not evaluated by Garin et al, while its psychometric evidence was confirmed in five regions with a total of 22 countries with 15 languages. However, this instrument is appropriate when the researcher tends to use a newer tool whose validity and reliability are accepted, although it fails to cover the environmental factors and body structures related to the ICF.

Since the content comparison based on the ICF provides valuable information about the content of instruments, it can provide significant information in the process of choosing the appropriate instrument. The comparison of instruments based on ICF allows us to evaluate the content validity of each instrument. Further, it is important to consider the psychometric properties of a measurement instrument in selecting the appropriate measurement instrument.^13^ Furthermore, the validity, reliability, and responsiveness of instruments should be compared when one of these instruments should be considered for implementing in research or clinical settings. A recent review article in the area of HF-specific HRQOL measurement instruments compared the entire instrument by focusing on psychometric properties. Each instrument was scored from 0 to 100 by four experts by using a standardized tool named Evaluating Measures of Patient-Reported Outcomes (EMPRO). The highest reliability score related to LVDQ (72.8). The reasonable scores for validity were related to CHFQ, KCCQ, and MLHFQ (54.4-76.4). The reviewers gave the highest score of sensitivity to change for KCCQ (94.4). Only CHFQ (50) and KCCQ (72.2) could gain adequate interpretability scores. Finally, the highest overall scores belonged to KCCQ (64.4), MLHFQ (60.7), and CHFQ (59.2).^8^ In content analysis, CHFQ does not cover the activity and participation, environmental factors, and body structures. KCCQ and MLHFQ had more coverage of ICF component, and their construct validity, reliability and responsiveness were confirmed. Thus, the best measurement instrument for assessing the HRQOL in HF is related to KCCQ and MLHFQ, respectively, according to psychometric properties and content comparison. Of course, some concerns were raised on the content validity OF MLHFQ and all relevant items.^29^ Furthermore, content comparison provides information on the instruments overlapping and heterogeneity with regard to body function, body structure, activities, as well as participation and environmental factors, which may help researchers and clinicians choose QOL measurement instruments for a specific clinical context or research question.^6^ According to the available information, this systematic review is the first content comparison in the field of cardiovascular diseases and linking to the ICF, which is considered as one of the limitations of the study in terms of comparing the results. Accordingly, it is suggested that other researchers can focus on the content comparison of QOL measurement instruments based on ICF for other cardiovascular diseases such as MI. Including the English instruments is regarded as another limitation of this systematic review. Since personal factors were not included in the latest version of ICF, the concepts such as lifestyle, personal life, and outlook on life could not be linked to ICF.

## Conclusion

In this systematic review, ten instruments were identified and the data were compared by using a systematic content analysis approach based on the ICF framework. Based on the results, ICF is considered as an appropriate framework for comparing the content of HRQOL measurement instruments in HF. According to the psychometric properties and content analysis approach based on the ICF framework, KCCQ and MLHFQ are regarded as the appropriate measurement instruments for assessing the HRQOL in HF, respectively.

## Competing interests

This review was not funded and there is no conflict of interest.

## Ethical approval

Not applicable.

## Acknowledgments

The authors would like to thank all working on this study.

## Supplementary Materials


Supplementary file 1 contains serach strategy and Tables S1-S4.

## References

[1] Mozaffarian D, Benjamin EJ, Go AS, Arnett DK, Blaha MJ, Cushman M (2016). Executive summary: heart disease and stroke statistics—2016 update: a report from the American Heart Association. Circulation.

[2] Navidian A, Mobaraki H, Shakiba M (2017). The effect of education through motivational interviewing compared with conventional education on self-care behaviors in heart failure patients with depression. Patient Educ Couns.

[3] Heo S, Lennie TA, Okoli C, Moser DK (2009). Quality of life in patients with heart failure: ask the patients. Heart Lung.

[4] Olano‐Lizarraga M, Oroviogoicoechea C, Errasti‐Ibarrondo B, Saracíbar‐Razquin M (2016). The personal experience of living with chronic heart failure: a qualitative meta‐synthesis of the literature. J Clin Nurs.

[5] McMurray JJ, Stewart S (2000). Epidemiology, aetiology, and prognosis of heart failure. Heart.

[6] Ware JE, Sherbourne CD (1992). The MOS 36-item short-form health survey (SF-36): I Conceptual framework and item selection. Med Care.

[7] Garin O, Ferrer M, Pont À, Rué M, Kotzeva A, Wiklund I (2009). Disease-specific health-related quality of life questionnaires for heart failure: a systematic review with meta-analyses. Qual Life Res.

[8] Garin O, Herdman M, Vilagut G, Ferrer M, Ribera A, Rajmil L (2014). Assessing health-related quality of life in patients with heart failure: a systematic, standardized comparison of available measures. Heart Fail Rev.

[9] Moshki MK, Abdoljavad Abdoljavad, Minaee S, Vakilian F (2018). Content Comparison of Health-Related Quality of Life Measures in Heart Failure Based on the International Classification of Functioning, Disability, and Health: A Systematic Review Protocol. J Tehran Heart Cent.

[10] Cieza A, Brockow T, Ewert T, Amman E, Kollerits B, Chatterji S (2002). Linking health-status measurements to the international classification of functioning, disability and health. J Rehabil Med.

[11] Terwee CB, Jansma EP, Riphagen II, de Vet HC (2009). Development of a methodological PubMed search filter for finding studies on measurement properties of measurement instruments. Qual Life Res.

[12] Velstra I-M, Ballert CS, Cieza A (2011). A systematic literature review of outcome measures for upper extremity function using the international classification of functioning, disability, and health as reference. PM R.

[13] Cieza A, Geyh S, Chatterji S, Kostanjsek N, Ustun B, Stucki G (2005). ICF linking rules: an update based on lessons learned. J Rehabil Med.

[14] Cieza A, Stucki G (2005). Content comparison of health-related quality of life (HRQOL) instruments based on the international classification of functioning, disability and health (ICF). Qual Life Res.

[15] Geyh S, Cieza A, Kollerits B, Grimby G, Stucki G (2007). Content comparison of health-related quality of life measures used in stroke based on the international classification of functioning, disability and health (ICF): a systematic review. Qual Life Res.

[16] Dunderdale K, Thompson DR, Beer SF, Furze G, Miles JN (2008). Development and validation of a patient-centered health-related quality-of-life measure: the Chronic Heart Failure Assessment Tool. Eur J Heart Fail.

[17] Mannheimer B, Andersson B, Carlsson L, Währborg P (2007). The validation of a new quality of life questionnaire for patients with congestive heart failure–an extension of the Cardiac Health Profile. Scand Cardiovasc J.

[18] Guyatt GH, Nogradi S, Halcrow S, Singer J, Sullivan MJ, Fallen EL (1989). Development and testing of a new measure of health status for clinical trials in heart failure. J Gen Intern Med.

[19] Green CP, Porter CB, Bresnahan DR, Spertus JA (2000). Development and evaluation of the Kansas City Cardiomyopathy Questionnaire: a new health status measure for heart failure. J Am Coll Cardiol.

[20] O’Leary C, Jones P (2000). The left ventricular dysfunction questionnaire (LVD-36): reliability, validity, and responsiveness. Heart.

[21] Rector T, Kubo S, Cohn J (1987). Patients’ self-assessment of their congestive heart failure Part 2: content, reliability and validity of a new measure, the Minnesota Living with Heart Failure Questionnaire. Heart Fail.

[22] Wiklund I, Lindvall K, Swedberg K, Zupkis RV (1987). Self‐assessment of quality of life in severe heart failure: an instrument for clinical use. Scand J Psychol.

[23] Valenti L, Lim L, Heller RF, Knapp J (1996 Feb). An improved questionnaire for assessing quality of life after acute myocardial infarction. Qual Life Res.

[24] Shabetai R (1983). Cardiomyopathy: How far have we come in 25 years, how far yet to go?. JACC.

[25] Oldridge N, Hofer S, McGee H, Conroy R, Doyle F, Saner H (2014 Jan). The HeartQoL: part II Validation of a new core health-related quality of life questionnaire for patients with ischemic heart disease. Eur J Prev Cardiol.

[26] Weigl M, Cieza A, Harder M, Geyh S, Amann E, Kostanjsek N (2003). Linking osteoarthritis-specific health-status measures to the International Classification of Functioning, Disability, and Health (ICF). Osteoarthritis Cartilage.

[27] Fitzpatrick R, Bowling A, Gibbons E, Haywood K, Jenkinson C, Mackintosh A, et al. A structured review of patient-reported measures in relation to selected chronic conditions, perceptions of quality of care and carer impact. National Center for Health Outcomes Development, University of Oxford. 2006.

[28] Stucki A, Cieza A, Schuurmans MM, Ustun B, Stucki G, Gradinger F (2008). Content comparison of health-related quality of life instruments for obstructive sleep apnea. Sleep Med.

[29] Dunderdale K. Health-related quality of life in chronic heart failure: Development and validation of a patient-centred health-related quality of life measure [thesis]. University of York: Department of Health Sciences; 2007.

