# Supplementary material for: The content comparison of health-related quality of life measures in heart failure based on the international classification of functioning, disability, and health: a systematic review
Source: J Cardiovasc Thorac Res. 2019 Aug 13;11(3):167–75. doi: 10.15171/jcvtr.2019.29 (PMC6759616; doi:10.15171/jcvtr.2019.29)
Supplement: Supplementary file 1 — contains serach strategy and Tables S1-S4. [file jcvtr-11-167-s001.pdf]

Supplementary file 1

Search Strategy

The following filters have been developed for use with PubMed to search for records relating to patient-reported outcome measurement
PubMed version (HR-PRO[tiab] OR HRPRO[tiab] OR HRQL[tiab] OR HRQoL[tiab] OR QL[tiab] OR QoL[tiab] OR quality of life[tw] OR life quality[tw] OR health index*[tiab] OR health indices[tiab] OR health profile*[tiab] OR health status[tw] OR ((patient[tiab] OR self[tiab] OR child[tiab] OR parent[tiab] OR carer[tiab] OR proxy[tiab]) AND ((report[tiab] OR reported[tiab] OR reporting[tiab]) OR (rated[tiab] OR rating[tiab] OR ratings[tiab]) OR based[tiab] OR (assessed[tiab] OR assessment[tiab] OR assessments[tiab]))) OR ((disability[tiab] OR function[tiab] OR functional[tiab] OR functions[tiab] OR subjective[tiab] OR utility[tiab] OR utilities[tiab] OR wellbeing[tiab] OR well being[tiab]) AND (index[tiab] OR indices[tiab] OR instrument[tiab] OR instruments[tiab] OR measure[tiab] OR measures[tiab] OR questionnaire[tiab] OR questionnaires[tiab] OR profile[tiab] OR profiles[tiab] OR scale[tiab] OR scales[tiab] OR score[tiab] OR scores[tiab] OR status[tiab] OR survey[tiab] OR surveys[tiab]))

Table S1. The linkage of concepts from Heart failure -specific HRQOL instruments to the ICF categories: component body functions

Frequency of ICF component		HF specific HRQL instruments										
body functions (b)		MLHFQ	KCCQ	CHFQ	LVD-36	QLQ-SHF	CHP-CHF	CHAT	Mac New	San Diego	Heart QOL	Total
		13	20	21	31	17	27	29	22	32	10	222
B1 MENTAL FUNCTIONS	b114 Orientation functions								2			2
	b1265 Optimism					1						1
	b1266 Confidence				1				3			4
	b1263 Psychic stability				1	1	1					3
	b1268Temperament and personality functions, other specified							1				1
	b1300 Energy level	6	3	6	7	5	3	2		1	3	36
	b1303 Craving									1		1
	b1309 Energy and drive functions, unspecified		1									1
	b1342 Maintenance of sleep				1					2		3
	b1343 Quality of sleep	1	1									2
	b1348 Sleep functions, other specified							1				1
	Sleep functions, unspecified (b1349)						1	1				2
	b144 Memory functions	1										1

	b1440 Short-term memory						1					1
	b152 Emotional functions	1	5	13	10	6	10	7	2		5	59
	b1520 Appropriateness of emotion								1			1
	b1521 Regulation of emotion					1						1
	b1522 Range of emotion		2	1		1			6			10
	b1528Emotional functions, other specified	1		1			1	5				8
	b160 Thought functions							1				1
	b1641 Organization and planning							1				1
	b1646 Problem-solving						1					1
	Higher-level cognitive functions, other specified (b1648)						3					3
b2-SENSORY FUNCTIONS AND PAIN	b2401 Dizziness									1		1
	b28011Pain in chest					1	1		1	9		12
	b28015 Pain in lower limb				1		1		1	1		4
	b28018 Pain in body part, other specified						1					1
B4- FUNCTIONS OF THE CARDIOVASCULAR,	b435 Immunological system functions				1							1

HAEMATOLOGICAL, IMMUNOLOGICAL AND RESPIRATORY SYSTEMS	b4400 Respiration rate	1	4		2	1	1	10	1	7	1	28
	Respiration functions, unspecified (b4409)		1									1
	b455 Exercise tolerance functions								1	1		2
	b4550 General physical endurance				3				3	7	1	14
	d4558 Moving around, other specified				1							1
	b469 Additional functions and sensations of the cardiovascular and respiratory systems, other specified and unspecified	1	3		1		1			1		7
B5- FUNCTIONS OF THE DIGESTIVE, METABOLIC AND ENDOCRINE SYSTEMS	b530 Weight maintenance functions									1		1
	b5350 Sensation of nausea				1							1
	b5358 Sensations associated with the digestive system, other specified						1					1
b6- GENITOURINARY AND REPRODUCTIVE FUNCTIONS	b640 Sexual functions	1										1

b 7- NEUROMUSCULOSKELETAL AND MOVEMENT-RELATED FUNCTIONS	b730 Muscle power functions				1				1			2

Table S2. The linkage of concepts from Heart failure-specific HRQL instruments to the ICF categories: component activity and participation

Frequency of ICF component		HF-specific HRQL instruments										
ACTIVITIES AND PARTICIPATION (d)		MLHFQ s	KCCQ s	CHFQ	LVD-36	QLQ-SHF	CHP-CHF s	CHAT s	Mac New	San Diego	Heart QOL	Total
		15	21		15	9	8	32	6	16	12	134
D1- learning and applying knowledge	d160 Focusing attention	1					1	1				3
	d177 Making decisions					1						1
D2- GENERAL TASKS AND DEMANDS	d2101Undertaking a complex task						1					1
	d2200 Carrying out multiple tasks				1							1
	d2203Undertaking multiple tasks in a group				1							1

	d2208 Undertaking multiple tasks, other specified						1					1
	d230 Carrying out daily routine					1		9				10
	d2301 Managing daily routine								1			1
	d240 Handling stress and other psychological demands						1					1
	Handling responsibilities(d2400)				1							1
	d2401Handling stress	1										1
d3- COMMUNICATION	d399 Communication, unspecified							1				1
d4. Mobility	d410 Changing basic body position		1									1
	d4300 Lifting										1	1
	d4301 Carrying in the hands		1								1	2
	d415 3Maintaining a sitting position		1									1
	d450 Walking	2						1				3
	d4500 Walking short distances		1			1				1		3
	d4501Walking long distance				1					1	1	3
	d4509 Walking, unspecified							2				2
	d455 Moving around				1				1			2
	d4551 Climbing	1	1			2				2	2	8
	d4552 Running		1									1
	Moving around, other specified (d4558)		1									1
	d4600 Moving around within the home					1						1
	d4602 Moving around outside the home and other buildings				1	2		1				4

	d469 Walking and moving, other specified and unspecified									3		3
	d470 Using transportation	1										1
	d475 Driving	1										1
d5- SELF-CARE	d510 Washing oneself				1							1
	d5101 Washing whole body		1									1
	d5109 Washing oneself, unspecified							2				2
	d540 Dressing		1									1
	d5400 Putting on clothes				1							1
	d5409 Dressing, unspecified							2				2
	d550 Eating							1				1
	d560 Drinking							1				1
	5701Managing diet and fitness		1									1
	d5708Looking after one's health, other specified		4									4
	d5709 Looking after one's health, unspecified	1										1
D6- DOMESTIC LIFE	d6200 Shopping							1		1		2
	d6209 Acquisition of goods and services, unspecified									1		1
	d6309 Preparing meals, unspecified							1				1
	d640 Doing housework		1		1					1		3
	d6402 Cleaning living area				1						1	2
	d6408 Doing housework, other specified	1	2								3	6
	d6409 Doing housework, unspecified							1				1
	d649Household tasks, other specified and unspecified					1						1
	d6505Taking care of plants, indoors and outdoors							1		1	1	3
	d6509 Caring for household objects, unspecified									4		4

d7- INTERPERSONAL INTERACTIONS AND RELATIONSHIPS	D710 Basic interpersonal interactions								2			2
	d750 Informal social relationships	1										1
	d7500 Informal social relationships with friends						1					1
	d760 Family relationships	1						1	1			3
	d7609 Family relationships, unspecified						1					1
	d770 Intimate relationships		1					1				2
	d7702 Sexual relationships						1		1	1		3
d8- MAJOR LIFE AREAS	d850 Remunerative employment	1										1
	d8502 Full-time employment							1				1
d9- COMMUNITY, SOCIAL AND CIVIC LIFE	d920 Recreation and leisure	1	2				1	1				5
	d9200Play				1							1
	d9201 Sports	1			1						2	4
	d9204 Hobbies	1	1					1				3
	d9205 Socializing				1			2				3
	d9208 Recreation and leisure, other specified				2							2

Table S3. The linkage of concepts from Heart failure-specific HRQOL instruments to the ICF categories: component environmental factors

Frequency of ICF component		HF-specific HRQL instruments										
ENVIRONMENTAL FACTORS (e)		MLHFQ	KCCQ	CHFQ	LVD-36	QLQ-SHF	CHP-CHF	CHAT	Mac New	San Diego	Hear QOL	Total
		6	4		2		2	5		9		28
	e1101 Drugs				1			2		3		6
	Products and technology for personal use in		2							3		5

E1. PRODUCTS AND TECHNOLOGY	daily living, other specified (e1158)											
	e1159 Products and technology for personal use in daily living, other specified									3		3
	E120 Products and technology for personal indoor and outdoor mobility and transportation				1							1
	e1650 Financial assets	1										1
E2. NATURAL ENVIRONMENT AND HUMAN-MADE CHANGES TO ENVIRONMENT	e2250 Temperature						1					1
	e2254 Wind						1					1
e3. support and relationship	e310 Immediate family	1	1									2
	e315 Extended family	1										1
	e320 Friends	1	1									2
	e355 Health professionals							2				2

		MLHFQ	KCCQ	CHFQ	LVD-36	QLQ-SHF	CHP-CHF	CHAT	Mac New	San Diego	Hear QOL	Total
e4 . ATTITUDES	e410Individual attitudes of immediate family members							1				1
E5. SERVICES, SYSTEMS AND POLICIES	e5800 Health services	2										2

Table S4. The linkage of concepts from Heart failure -specific HRQOL instruments to the ICF categories: component body structure

Frequency of ICF component		HF specific HRQL instruments										
BODY STRUCTURES (S)		MLHFQ	KCCQ	CHFQ	LVD-36	QLQ-SHF	CHP-CHF	CHAT	Mac New	San Diego	Hear QOL	Total
		2	7		1		2			9		21
S7- STRUCTURES RELATED TO MOVEMENT	s3200 Teeth									1		1
	s410 Structure of cardiovascular system									2		2
	s598 Structures related to the digestive, metabolic and endocrine systems, other specified									1		1
	s710 1Bones of face									1		1
	s7300 Structure of upper arm									1		1
	s7501Structure of lower leg	1	2		1		1			1		6

	s7502structure of ankle and foot	1	5			1			2		9
